# Wernicke Encephalopathy During Neoadjuvant Folinic Acid, Fluorouracil, and Oxaliplatin (FOLFOX) Therapy for Rectal Adenocarcinoma: A Case Report and Review of Diagnostic Challenges

**DOI:** 10.7759/cureus.114031

**Published:** 2026-08-05

**Authors:** Matthew Baron, Husam Abuhayyeh, Bushra Shariff

**Affiliations:** 1 Internal Medicine, Moffitt Cancer Center, Tampa, USA; 2 Neurology, University of South Florida, Tampa, USA

**Keywords:** 5‑fluorouracil, malnutrition, nystagmus, rectal adenocarcinoma, thiamine deficiency, vitamin b1 deficiency, wernicke encephalopathy

## Abstract

Wernicke encephalopathy (WE) is an acute neurologic disorder caused by thiamine deficiency. A 55-year-old Caucasian female patient undergoing treatment with neoadjuvant folinic acid, fluorouracil, and oxaliplatin for locally advanced rectal adenocarcinoma presented with intractable nausea and vomiting resulting in clinically significant weight loss and malnutrition. Oculomotor dysfunction and gait disturbance were noted, and empiric treatment for WE was initiated with high-dose intravenous thiamine. A contrast-enhanced MRI brain was obtained and showed no acute abnormality. Diagnosis was confirmed by therapeutic response to thiamine in conjunction with a low serum thiamine level.

## Introduction

Thiamine (vitamin B1) is a vital coenzyme involved in metabolism and neurotransmitter synthesis [1]. Wernicke encephalopathy (WE) is an acute neurologic disorder caused by thiamine deficiency and classically characterized by the triad of altered mental status, eye movement abnormalities, and unsteady gait. WE is a rare condition with an estimated clinical prevalence of approximately 0.1% in the general population, but with postmortem analysis suggesting true prevalence may be as much as fivefold higher [2]. Although most commonly associated with alcohol use disorder, WE is increasingly recognized in nonalcoholic populations, including patients with cancer. Cancer-related risk factors include malnutrition, increased metabolic demands, and chemotherapy-related alterations in thiamine function [3,4]. Diagnosis can be challenging because neurologic manifestations are often subtle or incomplete, the classic triad is present in only a minority of cases, and neuroimaging may be normal [3,5]. Consequently, delayed recognition remains common and may result in irreversible neurologic injury or death. We describe a case of WE developing during neoadjuvant folinic acid, fluorouracil, and oxaliplatin (FOLFOX) chemotherapy for rectal adenocarcinoma and review the diagnostic challenges and clinical implications of thiamine deficiency in oncology patients.

## Case presentation

A 55-year-old Caucasian female patient presented to the emergency room of our tertiary care hospital with complaints of nausea, vomiting, poor oral intake, weight loss, and fatigue waxing and waning over the last two months. Her past medical history was significant for stage III rectal adenocarcinoma undergoing neoadjuvant treatment status post five cycles of FOLFOX followed by fluorouracil and leucovorin alone for cycle 6 due to poor tolerance. She tolerated the first three cycles of chemotherapy well, but experienced progressive symptoms with the next three cycles, with symptoms waxing and waning in relation to each cycle. The patient was seen in the medical oncology clinic two weeks after completion of cycle 6 and referred for hospital admission for further investigation and treatment of symptoms atypical in both duration and intensity.

Patient described mucositis after the last couple of cycles of chemotherapy, which had resolved by the time of presentation. She attributed her ongoing nausea, vomiting, and poor oral intake to ongoing dysgeusia. Her fatigue was described as persistent tiredness and lack of energy without focal weakness, dyspnea, or chest discomfort. The patient reported episodes of dizziness after cycles 4 and 5, respectively, which were worked up at urgent care and deemed secondary to dehydration. She reported progressive diarrhea associated with each cycle of chemotherapy, which had resolved by the time of presentation. Her weight loss was reported to be minimal with the first three cycles of treatment and then became precipitous thereafter. The patient described some mild cognitive impairment characterized as brain fogginess, without overt memory loss, disorientation, or hallucinations. She denied fever, abdominal pain, dysuria, vision changes, or recent falls.

On physical examination, the oral cavity had multiple large areas of dental caries without associated mucosal tenderness, induration, or fluctuance. She appeared euvolemic after receiving a 1 L bolus of normal saline in the emergency department. The patient was alert and oriented to person, place, time, and situation. Cranial nerves II-XII were grossly intact. She was able to move all extremities against gravity and without loss of sensation to light touch. Finger-to-nose testing was normal. The patient demonstrated wide-based stance, but ambulation was not attempted due to physical deconditioning and malaise. Review of documented weights demonstrated a prechemotherapy weight of 109 kg, falling slightly to 103 kg after the third cycle of chemotherapy, and then dropping precipitously to 84 kg on admission, resulting in a 23% weight loss. The remainder of the physical examination was within normal limits.

Initial laboratory evaluation is described in Table 1. Complete blood count demonstrated pancytopenia following treatment with fluorouracil and leucovorin two weeks prior to presentation. All cell lines slowly improved throughout hospitalization, with resolution of neutropenia at the time of discharge. Complete metabolic panel demonstrated hypokalemia in the setting of vomiting and hypoalbuminemia in the setting of subacute weight loss.

**Table 1:** Initial laboratory findings Laboratory findings demonstrate pancytopenia in the setting of recent cytotoxic chemotherapy

Test	Results	Units	Normal range
White blood cells	1.70	k/mcL	4.0-10.9
Absolute neutrophil count	0.80	k/mcL	1.8-7.8
Hemoglobin	9.2	g/dL	13.4-16.9
Platelet count	123	k/mcL	143-382
Sodium	139	mmol/L	134-145
Potassium	2.9	mmol/L	3.4-4.5
Chloride	102	mmol/L	96-107
Bicarbonate	25	mmol/L	22-30
Creatinine	0.51	mg/dL	0.7-1.3
Blood urea nitrogen	6	mg/dL	6-23
Glucose	83	mg/dL	70-110
Albumin	3.3	g/dL	3.5-5.2
Calcium corrected	9.3	mg/dL	8.6-10.2
Magnesium	1.7	mg/dL	1.6-2.3
Alkaline phosphatase	70	U/L	40-130
Aspartate aminotransferase	24	U/L	5-34
Alanine aminotransferase	14	U/L	0-55
Total bilirubin	0.7	mg/dL	0.0-1.2

Patient’s nausea and vomiting showed improvement over the first 48 hours of admission with conservative management including intravenous (IV) hydration and antiemetics. She was advanced from clear liquid, to full liquid, to regular diet. On day 3 of hospitalization, the patient reported persistent disequilibrium described as a rocking-boat sensation over the past week, associated with dizziness worse with lateral gaze, despite improving nausea. Focused physical exam was significant for sluggishly reactive pupils bilaterally with lateral-gaze rotational nystagmus at subextreme lateral gaze bilaterally, with a rebound component when looking back to primary gaze. She also had frequent saccadic intrusions. Cranial nerve exam was otherwise intact. Finger-to-nose testing remained intact. High-dose thiamine at 500 mg three times daily was initiated secondary to concern for WE in the setting of newly appreciated ocular findings. Contrast-enhanced MRI brain was obtained and demonstrated no acute abnormality. Vitamin B1 level was obtained prior to initiation of IV thiamine, but treatment was initiated empirically based on clinical findings prior to the result. Following discharge, vitamin B1 level returned low at 34 nmol/L with a laboratory-reported reference range of 70-180 nmol/L. The patient received six total doses of IV thiamine for a total of 3,000 mg with improving nystagmus. Gait remained wide-based, but the patient demonstrated functional improvement with the ability to ambulate 360 ft without assistance on the day of discharge. She was discharged home on hospital day 5 with a script for oral thiamine 100 mg daily.

## Discussion

WE is an acute neuropsychiatric syndrome caused by thiamine (vitamin B1) deficiency, classically characterized by the triad of encephalopathy, oculomotor dysfunction, and gait ataxia. Clinical studies suggest a prevalence of WE within the general population of approximately 0.1%, but autopsy results suggest this is an underestimate, with upward of 80% of cases missed by routine exam and evaluation [2]. Nonalcoholic WE is increasingly recognized in patients with cancer and other conditions associated with malnutrition or increased metabolic demand. In patients with malignancy, the diagnosis remains challenging because the presentation often overlaps with treatment-related toxicities, and clinicians may have a lower index of suspicion in the absence of significant alcohol use. This case highlights the importance of considering thiamine deficiency in oncology patients who develop significant malnutrition as a result of their gastrointestinal symptoms resulting from cytotoxic therapy.

Several factors likely contributed to thiamine depletion in our patient. Prior to admission, she experienced prolonged nausea, vomiting, diarrhea, dysgeusia, and poor oral intake during neoadjuvant treatment with FOLFOX. These complications resulted in rapid weight loss of approximately 25 kg over a relatively short period. Cancer itself further increases susceptibility to thiamine deficiency through increased metabolic demand and utilization by rapidly proliferating tissues [3]. In addition, the 5-fluorouracil metabolite fluoroacetate may inactivate thiamine-dependent enzymes, producing a functional thiamine deficiency, even when circulating thiamine levels remain close to normal [4]. Taken together, the combination of reduced intake, gastrointestinal losses, cancer-related metabolic stress, and fluoropyrimidine exposure created a substantial risk for clinically significant thiamine depletion in this patient.

A notable feature of this case was the absence of the complete classic triad of WE at presentation. Systematic review suggests that only 30% of cancer patients ultimately diagnosed with WE exhibited the entire triad [3]. Reliance on the classical triad alone therefore contributes to underdiagnosis and delayed treatment. The modified Caine et al.'s criteria propose diagnosis when at least two of the following are present: nutritional deficiency, ocular abnormalities, cerebellar dysfunction, and altered mental status [6]. Caine et al. were able to achieve 100% sensitivity for the diagnosis of WE within their study population utilizing these modified criteria. Our patient demonstrated only two of the classic symptoms, but met the modified Caine criteria with nutritional deficiency, ocular abnormalities, and wide-based gait. This case supports application of the broader diagnostic framework in high-risk populations.

The role of neuroimaging in diagnosing WE also deserves emphasis. Contrast-enhanced brain MRI obtained in our patient was normal and unchanged from a baseline study done a month prior despite a low serum thiamine level and clinical response to thiamine replacement. Although characteristic MRI abnormalities comprising lesions within the thalamus, periaqueductal area, tectum, mammillary bodies, hypothalamus, or periventricular gray matter of the fourth ventricle may support the diagnosis, imaging lacks sufficient sensitivity to exclude WE, with meta-analysis suggesting a sensitivity of around 82% for even a single typical lesion [5]. Similarly, measurement of serum thiamine concentrations may support the diagnosis but should not delay treatment, as laboratory results are frequently not immediately available and, in our case, did not result until after discharge more than a week later.

Prompt treatment is critical because delayed recognition can lead to permanent neurologic disability or death. Literature and guideline review do not demonstrate consensus on optimal IV thiamine dosing, but the 500 mg IV three times daily dosing utilized in our case is the most common, with doses at or above this level tending toward earlier and more robust improvement [7]. Concerns regarding treatment toxicity should not deter empiric treatment, as IV thiamine possesses an excellent safety profile with major reactions affecting less than 0.01% of the treatment population [8].

## Conclusions

In conclusion, this case illustrates a reversible but potentially life-threatening complication of fluoropyrimidine-based chemotherapy. Patients with cancer frequently possess multiple risk factors for thiamine deficiency, including inadequate nutritional intake, gastrointestinal losses, increased metabolic demands, and direct chemotherapy-related effects on thiamine metabolism. Clinicians should maintain a high index of suspicion for WE in oncology patients presenting with substantial weight loss, cognitive changes, oculomotor abnormalities, or gait disturbances, even in the absence of alcohol use disorder or characteristic MRI findings.

WE is a clinical diagnosis. Symptoms may sometimes be subtle and obscured by the physical deconditioning and malaise frequently seen in our cancer population. Despite increasing medical specialization, especially at our dedicated cancer centers, a basic neurologic exam is not beyond the purview of the general internist. Unfortunately, postmortem analysis suggests that failure to diagnose is common. Early recognition and prompt administration of high-dose IV thiamine may prevent permanent neurologic sequelae and significantly improve patient outcomes. IV thiamine is a low-risk medication, and empiric treatment should be initiated while differential diagnoses are narrowed with further clarifying workup.
